# Celiac Disease Presenting with Peripheral Neuropathy in Children: A Case Report

**DOI:** 10.3390/ijerph14070785

**Published:** 2017-07-14

**Authors:** Alessandra Pacitto, Alessandra Paglino, Lorenza Di Genova, Alberto Leonardi, Edoardo Farinelli, Nicola Principi, Giuseppe di Cara, Susanna Esposito

**Affiliations:** 1 Pediatric Clinic, Università degli Studi di Perugia, 06132 Perugia, Italy; alessandra-pacitto@libero.it (A.P.); apaglino@yahoo.it (A.P.); lory.digenova@gmail.com (L.D.G.); alberto.leonardi88@gmail.com (A.L.); edoardo.farinelli@gmail.com (E.F.); giuseppe.dicara@unipg.it (G.d.C.); 2 Pediatric Highly Intensive Care Unit, Fondazione IRCCS Ca’ Granda Ospedale Maggiore Policlinico, Università degli Studi di Milano, 20122 Milan, Italy; nicola.principi@unimi.it

**Keywords:** atypical celiac disease, celiac disease, gluten-free diet, Guillain-Barrè syndrome, peripheral neuropathy

## Abstract

**Background:** Clinically relevant neurological manifestations in children with celiac disease (CD) are unusual, especially when they are considered as signs of the onset of the disease. In this paper, a case of Guillain-Barrè syndrome (GBS) as the first manifestation of CD in a 23-month-old child is reported. **Case presentation:** We describe a case of CD onset with peripheral neuropathy in a 23-month-old Bulgarian boy presenting with a sudden refusal to walk and absence of deep tendon reflexes in both lower limbs. Neurological symptoms were preceded by two months of gastrointestinal symptoms such as vomiting, abdominal distention, and clear signs of malnutrition and weight loss. When we evaluated the child six months after the onset of the symptoms, clinical and laboratory findings showed clear signs of peripheral neuropathy associated with malnutrition. Serum deamidated gliadin and tissue transglutaminase antibodies were therefore measured. The anti-gliadin levels were more than sixteen times higher than normal and the IgA anti-transglutaminase levels were four times higher than normal. Anti-endomysium antibodies were positive, and human leukocyte antigens (HLA) II typing confirmed a genetic predisposition to CD (DQ2 positive and DQ8 negative). Given the association between the clinical evidence of the disease and the results of the celiac screening tests, a diagnosis of CD was made without biopsy confirmation of the enteropathy. The child began a restricted gluten-free diet that led to complete recovery of the peripheral neuropathy, walking, reflexes, and overall improvement after three months on the diet. **Conclusion:** Our case underlines the rare but possible associations between CD and peripheral neuropathy in children as an onset symptom, even in the absence of gastrointestinal manifestations, thus suggesting that CD should always be considered in the differential diagnosis of peripheral neuropathy in children. A good knowledge of the extra-intestinal manifestations of CD is essential for the rapid introduction of a gluten-free diet that could be useful for the resolution of the neurological symptoms.

## 1. Background

Celiac disease (CD) is a systemic inflammatory immune-mediated disorder triggered by the ingestion of gluten in genetically susceptible individuals. In asymptomatic patients, the overall prevalence of CD is approximately 1% of the general population [1]. For years, CD was thought to only be a gastrointestinal disease mainly characterized by severe malabsorption associated with chronic diarrhoea, abdominal bloating, steatorrhea, and failure to thrive. However, beginning in the 1960s, several studies have demonstrated that CD might present with extra-intestinal manifestations, which may precede and/or accompany gastrointestinal symptoms or occur without any gastrointestinal problems [2].

Neurologic and psychiatric disorders are among the most common extra-intestinal manifestations in adults with CD, and cerebellar ataxia, epilepsy, and peripheral neuropathy are those more frequently described [3]. In adults with CD, the prevalence of neurological complications has been estimated to be as high as 36% [4,5]. In contrast, the association between a clinically evident neurologic disease and CD in children is rare. A recent study by Lionetti et al. showed that children with CD have no increased risk of epilepsy development [6]. Moreover, although in most studies the incidence of headache, cerebella dysfunction, and brain white matter lesions was found to be slightly higher in CD children than in healthy controls, the rates were significantly lower than those reported for adults. The incidence of peripheral neuropathy in association with CD in patients of pediatric and adult age is only 0.1% and 7.4%, respectively. In this paper, a case of Guillain-Barrè syndrome (GBS) as the first manifestation of CD in a 23-month-old child is reported.

## 2. Case Presentation

A 23-month-old, male, Bulgarian child was admitted to our hospital, owing to worsening of general conditions, unsteady walking, and the association of neurological symptoms. His birth weight was in the normal range, and no neonatal problems had been reported. His physical, cognitive, communicative, and socio-emotional development milestones were regularly reached. There was no history of neuromuscular congenital or autoimmune disease in his family except for in a 50-year-old aunt with autoimmune thyroiditis.

A personal history evaluation revealed that the child had been hospitalized in Bulgaria twice. The first time, six months before our admission, was due to a sudden refusal to walk associated with acute weakness of both legs and loss of deep tendon reflexes. Electromyography showed an axonal demyelinating injury of the distal nerves of the lower limbs, and lumbar puncture showed protein-cytological dissociation in the cerebrospinal fluid (white blood cells, 16 cells/µL; proteins, 910 mg/dL). On the basis of these clinical and laboratory findings, GBS was diagnosed, and intravenous immunoglobulins (500 mg/kg/day i.v.) were given for five days. This treatment resulted in a mild improvement in walking, although the deep tendon reflexes were still depressed. Reflexes of the upper limbs were preserved during the entire treatment.

Two months later, he experienced a new episode characterized by a new sudden walking refusal associated with a 1.5-kg weight loss during this period. He was hospitalized again in Bulgaria, and no diagnostic examination was performed. An anti-inflammatory therapy with ibuprofen and a cow’s milk protein-free diet was prescribed. After discharge, he still did not walk, was irritable and showed gastrointestinal symptoms such as vomiting, loss of appetence, and the presence of stools containing undigested food.

**Figure 1:**
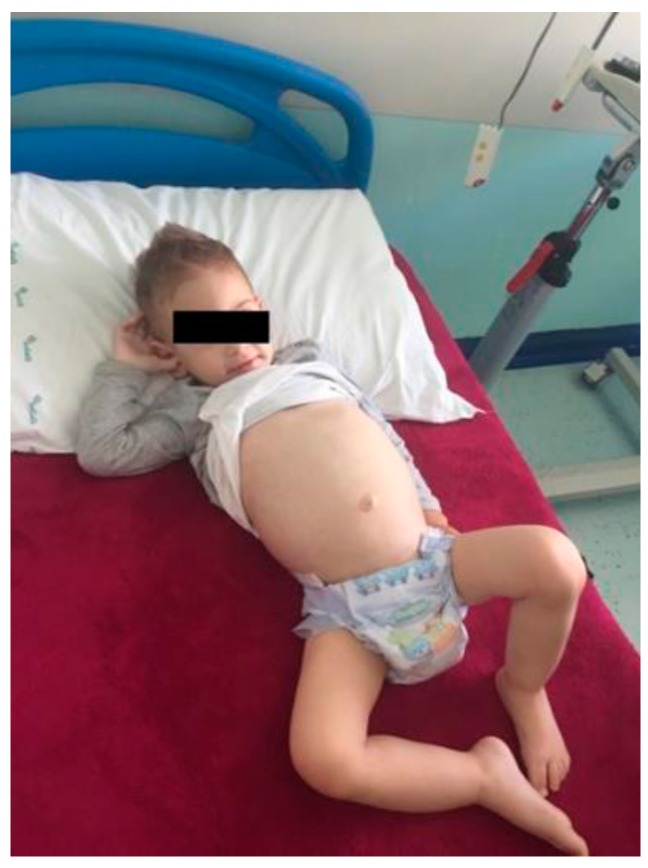
A 23-month-old child at admission six months after the onset of Guillain-Barré syndrome (GBS) appeared pale and mildly dehydrated, with scarce adiposity and muscle hypotrophy. He was unable to walk and had normal mental status, low muscle tone, absence of deep tendon reflexes in both legs, normal strength and sensorial evaluation of the upper limbs, and no deficit of the cranial nerves, nor cerebellar or meningeal signs. The Babinski sign was bilaterally absent, and the child had cold, swollen ankles and an abdomen severely distended with active peristalsis.

The child was brought to our attention six months after the onset of GBS. Examination revealed an irritable, pale, and mildly dehydrated child with scarce adiposity and muscle hypotrophy. He was unable to walk. His weight was 11.3 kg (30th percentile), his height was 86.5 cm (45th percentile), and his head circumference was 46 cm (10th percentile). Growth curves showed a stunting from the beginning of his disorders. Neurological examination documented a normal mental status, decreased muscle tone, and the absence of deep tendon reflexes in both legs. Strength and sensorial evaluation of the upper limbs was normal. There were no cranial nerve deficits, nor cerebellar or meningeal signs. The Babinski sign was bilaterally absent. He had cold, swollen ankles. His abdomen was severely distended, with lively peristalsis (Figure 1).

The initial work-up showed low levels of pre-albumin (13.4 mg/dL) and albumin (2 g/dL) as well as increased liver enzymes (SGOT 120 UI/L, SGPT 76 UI/L) and an altered coagulation framework (PT 26.2 s, INR 2.23, ratio 2.17). He was not anemic and had normal levels of ferritin and serum iron, and a normal transferrin saturation index. Blood fat-soluble vitamins (i.e., vitamin D, vitamin E, and vitamin K) were extremely low. Vitamin B12 and folic acid levels were normal. Intramuscular vitamin K supplementation was performed with rapid normalization of liver function and coagulation function. Vitamin D oral replacement therapy and physiotherapy were also started. Electromyography was not repeated because of the lack of parental consent. Considering the clinical features, peripheral neuropathy, gastrointestinal symptoms, and laboratory findings, the child was finally assessed for CD. The deamidated gliadin protein antibody (AGA) levels were 164 U/mL (normal values, <10 U/mL), transglutaminase levels were 46 U/mL (normal values, <10 U/mL), and he was positive for anti-endomysium antibodies (EMAs). Human leukocyte antigens (HLA) II typing confirmed a genetic predisposition to CD (DQ2 positive and DQ8 negative). According to the latest European Society for Paediatric Gastroenterology Hepatology and Nutrition (ESPGHAN) Guidelines [7], a duodenal biopsy was not performed because he was highly symptomatic with anti-EMA IgA- and HLA DQ2-positive with IgG anti-AGA and IgA anti-transglutaminase levels largely higher than the cut-off values. Fecal exams resulted negative for viruses and bacteria, including *Campylobacter jejuni*.

CD was diagnosed, and the child began a gluten-free diet and subsequently experienced a progressive recovery of walking, general clinical status improvement, and significant decrease in abdominal distention. Deep tendon reflexes were weak bilaterally. He went back to Bulgaria after one month of hospitalization and the administration of a gluten-free diet. HLA typing was suggested for first-degree relatives.

Table 1 summarizes the main clinical and laboratory findings in the study child according to his age.

**Figure 2:**
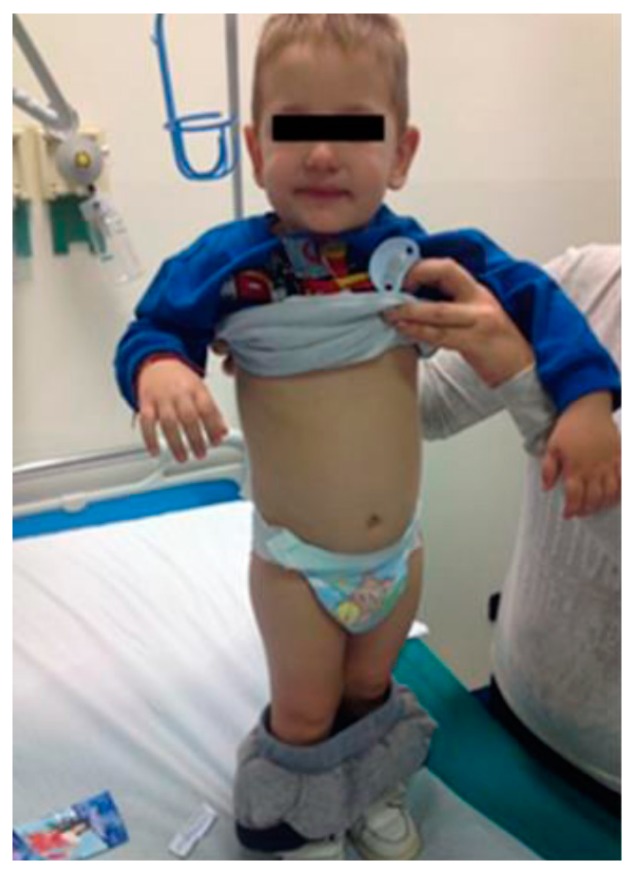
At the follow-up after three months of a gluten-free diet, at 27-months of age, the child was able to walk independently, was no longer irritable, and had no gastrointestinal symptoms; his skin appeared normally hydrated, and he had well represented adipose tissue and a plain abdomen.

At the follow-up, after three months of the gluten-free diet, at 27 months of age, the child was found to be able to walk independently, was no longer irritable, and had no gastrointestinal symptoms. Deep tendon reflexes were normally evoked in both legs. There was a complete recovery in his weight and length. His weight was in the 90th percentile, his length was in the 75th percentile, and his cranial circumference was in the 50th percentile. After general examination, his skin appeared normally hydrated, with well represented adipose tissue and a plain abdomen (Figure 2). The ankle edema had resolved. The nutritional panel was normal: pre-albumin was 20.4 md/dL and albumin 4.5 g/dL. Liver function and vitamin values were in a normal range.

## 3. Discussion

In the child reported here, recurrent episodes of GBS occurred several months before the development of classic gastrointestinal signs and symptoms of CD, thus highlighting the possibility that neurologic manifestations may precede gastrointestinal CD manifestations in children as well as adults. This observation has been reported in pediatric patients with headache [8,9], opsoclonus-myoclonus [10], recurrent optic neuritis [11], recurrent acute encephalopathy [12], status epilepticus [13,14], recurrent transient hemiplegia [15], cerebral venous sinus thrombosis [16], and transverse myelitis [17].

An increased risk of neuropathy in patients with CD has been reported in adults [18]. However, only one case of GBS preceding the gastrointestinal manifestation of CD has been previously described in a patient of pediatric age [19]; this lack of reports may explain why the diagnosis of CD was delayed and made only after a well-defined picture of malabsorption was clinically evident. Recent studies have shown a significant correlation between anti-ganglioside antibodies and neurological disorders in patients with underlying CD [19,20,21]. Gangliosides are glycosphingolipids that are abundant in the nervous system and in other tissues, including the gastrointestinal tract. It is not known what triggers the release of anti-ganglioside antibodies in people with gluten sensitivity. However, the mechanism is likely to involve the intestinal immune system response to ingested gliadin, a component of wheat gluten. Anti-ganglioside antibody formation in CD may play a role not only in developing neurological complications of CD patients, but also in developing CD itself. Unfortunately, we did not have any data on anti-ganglioside antibody formation in this case report.

However, GBS is a very rare disease in pediatric populations, particularly in younger children. In children <15 years of age, the incidence of GBS ranges from 0.5 to 1.5 cases per 100,000 population per year, with the lowest values in children <2 years old [20,21]. GBS is an autoimmune disease apparently triggered by an infection or by immunization. These factors may trigger an autoimmune response through molecular mimicry in which the host generates an immune response to an infectious organism which shares a ganglioside-like epitope with the host’s peripheral nervous system. Among the bacterial organisms that have a role in the development of GBS, *Campylobacter jejuni* has been best studied, showing that about 25% of patients with GBS have had a recent *Campylobacter jejuni* infection [21]. Now, it is well established that lipo-oligosaccharides located in the wall of *Campylobacter jejuni* cross-react with gangliosides in the axonal membrane of neurons. In our patient, no known triggering factor was reported, thus suggesting a role of gluten sensitization in the pathogenesis of the disease. The role of gluten sensitization appeared to be confirmed by the complete regression of the neurological symptoms and the rapid recovery of the general condition that occurred within a few months of a gluten-free diet. However, the pathophysiology of celiac neuropathy is still poorly understood and is probably multifactorial, as evidenced by the poor response to the gluten-free diet in several patients [22]. The most recent hypothesis suggests that different manifestations of gluten sensitivity depend on the role of transglutaminase antibodies in the humoral immune response. Different transglutaminases may lead to lesions in different body sites [23]. Transglutaminase 2 is considered the autoantigen in classic intestinal CD, whereas TG3 has a role as the autoantigen in dermatitis herpetiformis, and transglutaminase 6 seems to be important in brain damage. However, in other cases, malabsorption per se, leading to vitamin and micronutrient deficiency, may induce tissue damage [23].

The effect of the gluten-free diet on peripheral neuropathy and other neuromuscular disorders associated with CD is unclear. In only a few reports was the gluten-free diet was effective, whereas other studies indicated the persistence and progression of neuropathy despite an adequate gluten-free diet [8,9,10,11,12,13,14,15,16,17,18,19]. Although in our patient the gluten-free diet was effective, further studies are needed to assess the effect of the gluten-free diet and immunomodulation on these disorders and to investigate the underlying mechanisms of nervous system involvement associated with gluten sensitivity.

## 4. Conclusions

Our case underlines the rare but possible associations between CD and peripheral neuropathy in children as the onset symptom, even in the absence of gastrointestinal manifestations. Clinical data must be considered in order to recommend a gluten-free diet, which had a decisive result in our case. Further studies should be performed to clarify the etiology and prognosis of neurological manifestations in CD.

## Figures and Tables

**Table 1:** Main clinical and laboratory findings in the study child according to his age.

Data	Birth	17 Months of Age	19 Months of Age	23 Months of Age (Admission in Our Unit)
Clinical history	No problem	Sudden refusal to walk, acute weakness of both legs, loss of deep tendon reflexes	New sudden walking refusal, 1.5-kg weight loss	Inability to walk, irritability, pale, mild dehydration, scarce adiposity, muscle hypotrophy, growth curves exhibiting stunting, decreased muscle tone, absence of deep tendon reflexes in both legs, cold swollen ankles, severely distended abdomen with lively peristalsis
Electro-myography	Not performed	Axonal demyelinating injury of the distal nerves of the lower limbs	Not performed	Not repeated because of lack of parental consent
Other abnormal exams	Not performed	Protein-cytological dissociation in the cerebrospinal fluid	Not performed	Low levels of pre-albumin (13.4 mg/dL) and albumin (2 g/dL), increased liver enzymes (SGOT 120 UI/L, SGPT 76 UI/L), an altered coagulation framework (PT 26.2 s, INR 2.23, ratio 2.17), low blood fat-soluble vitamins (i.e., vitamin D, vitamin E, and vitamin K), positive deamidated gliadin protein antibody (AGA) levels (164 U/mL; normal values, <10 U/mL), positive transglutaminase antibodies (46 U/mL; normal values, <10 U/mL), positive anti-endomysium antibodies, human leukocyte antigens (HLA) II DQ2 positivity
Diagnosis	Normal newborn	GBS	Acute infection	Celiac disease with peripheral neuropathy
Therapy	None	Intravenous immunoglobulins for five days	Ibuprofen, cow’s milk protein-free diet	Intramuscular vitamin K supplementation, gluten-free diet
Outcome	Not applicable	Mild improvement in walking with deep reflexes still depressed	Refusal to walk, irritability, gastrointestinal symptoms (i.e., vomiting, loss of appetence, stools with undigested food)	Complete recovery in weight and length, normal ability to walk, absence of irritability, normal hydration, well represented adipose tissue, plain abdomen, absence of ankle edema, normal nutritional panel
